# Supplementary material for: RAC1-Amplified and RAC1-A159V Hotspot-Mutated Head and Neck Cancer Sensitive to the Rac Inhibitor EHop-016 In Vivo: A Proof-of-Concept Study
Source: Cancers (Basel). 2025 Jan 23;17(3):361. doi: 10.3390/cancers17030361 (PMC11816149; doi:10.3390/cancers17030361)
Supplement: Supplementary file 1 [file cancers-17-00361-s001.zip › File S1-westernblot.pptx]

## Slide 1
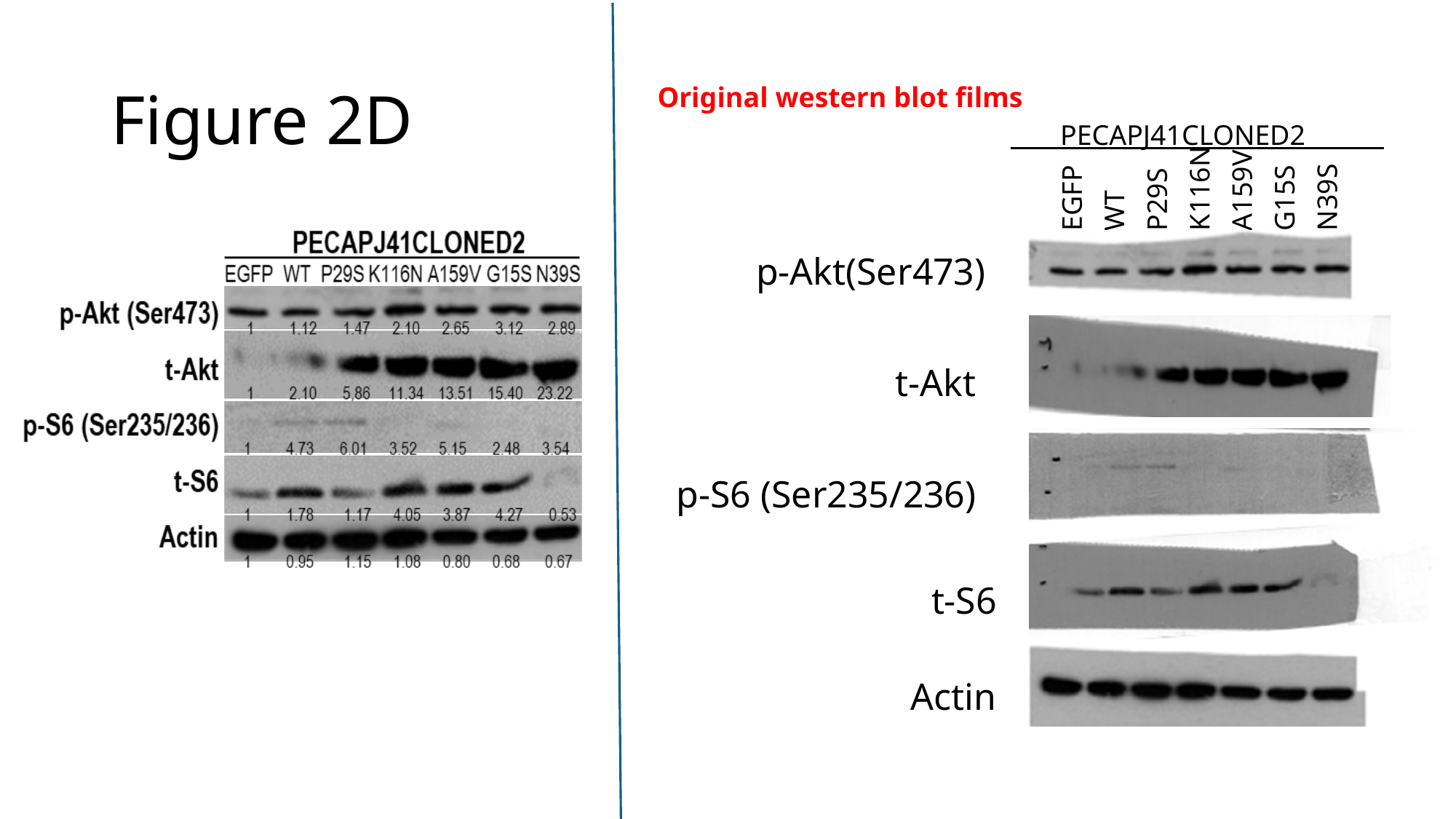

EGFP
WT
P29S
K116N
A159V
G15S
N39S
# Figure 2D
Original western blot films
 PECAPJ41CLONED2
p-Akt(Ser473)
t-Akt
p-S6 (Ser235/236)
t-S6
Actin

## Slide 2
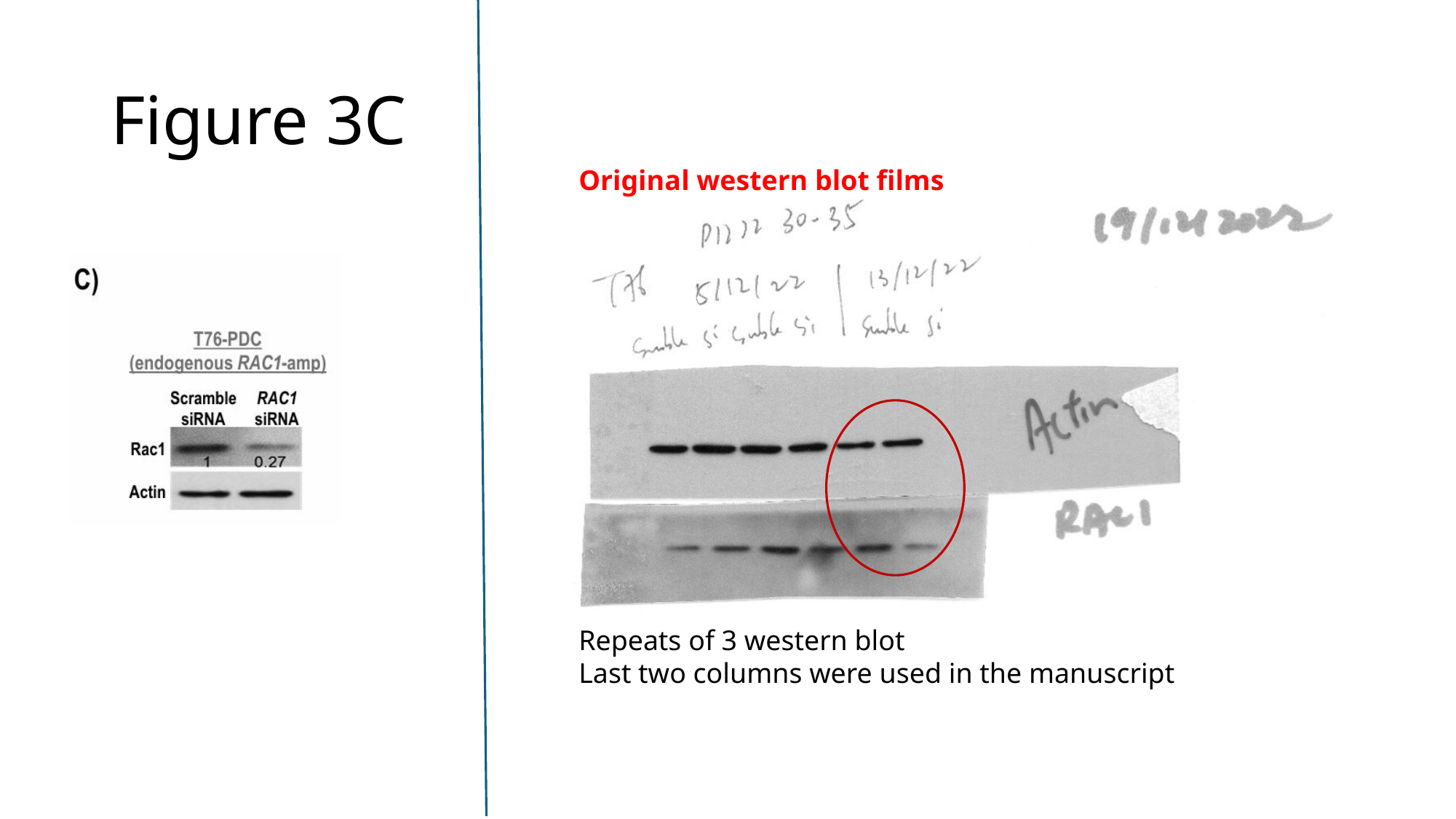

# Figure 3C
Original western blot films
Repeats of 3 western blot
Last two columns were used in the manuscript

## Slide 3
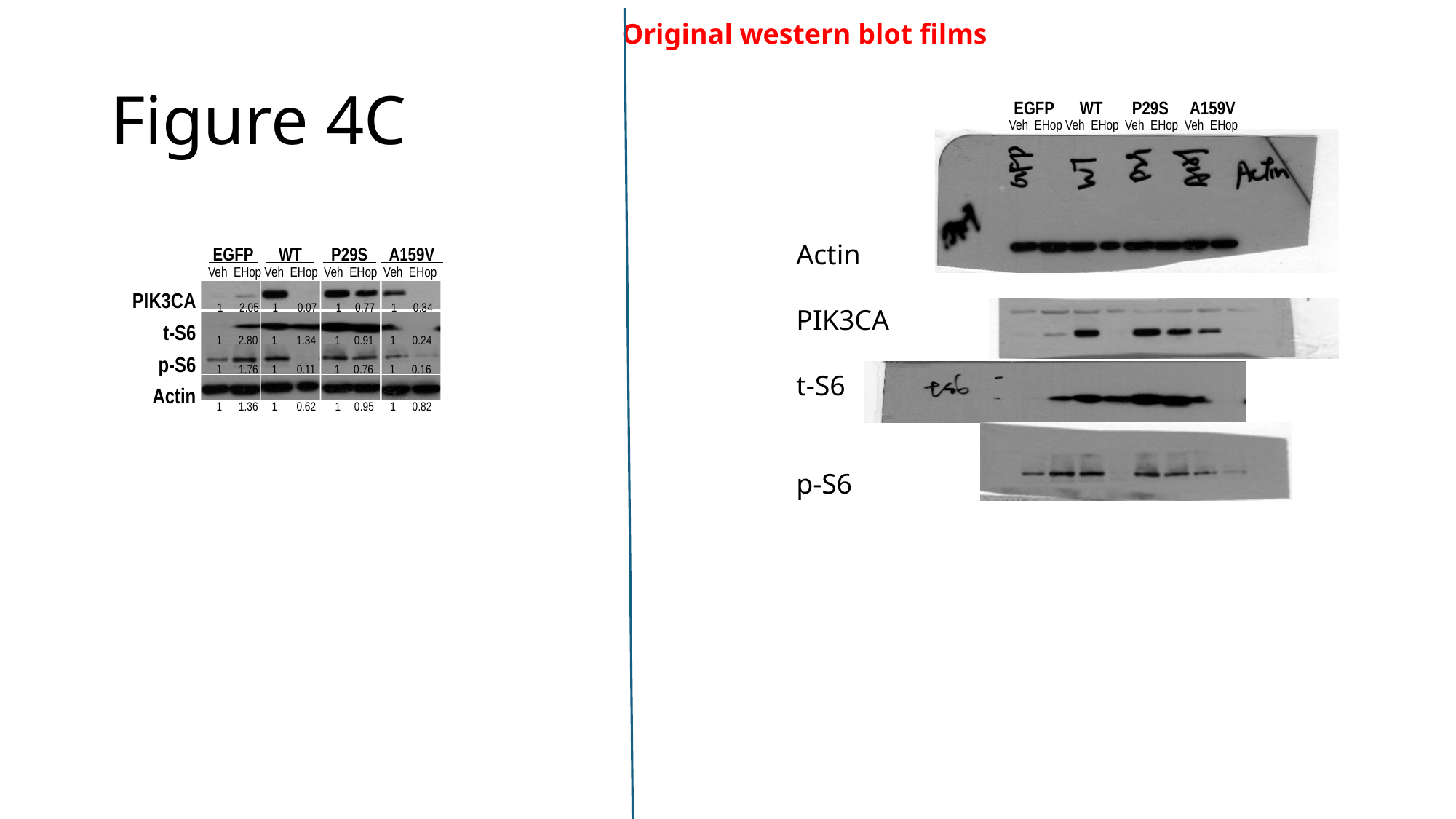

Original western blot films
# Figure 4C
 EGFP
 WT
 P29S
 A159V
Veh EHop Veh EHop Veh EHop Veh EHop
Actin
PIK3CA
t-S6
p-S6
 EGFP
 WT
 P29S
 A159V
Veh EHop Veh EHop Veh EHop Veh EHop
PIK3CA
t-S6
p-S6
Actin
1 2.05 1 0.07 1 0.77 1 0.34
1 2.80 1 1.34 1 0.91 1 0.24
1 1.76 1 0.11 1 0.76 1 0.16
1 1.36 1 0.62 1 0.95 1 0.82

## Slide 4
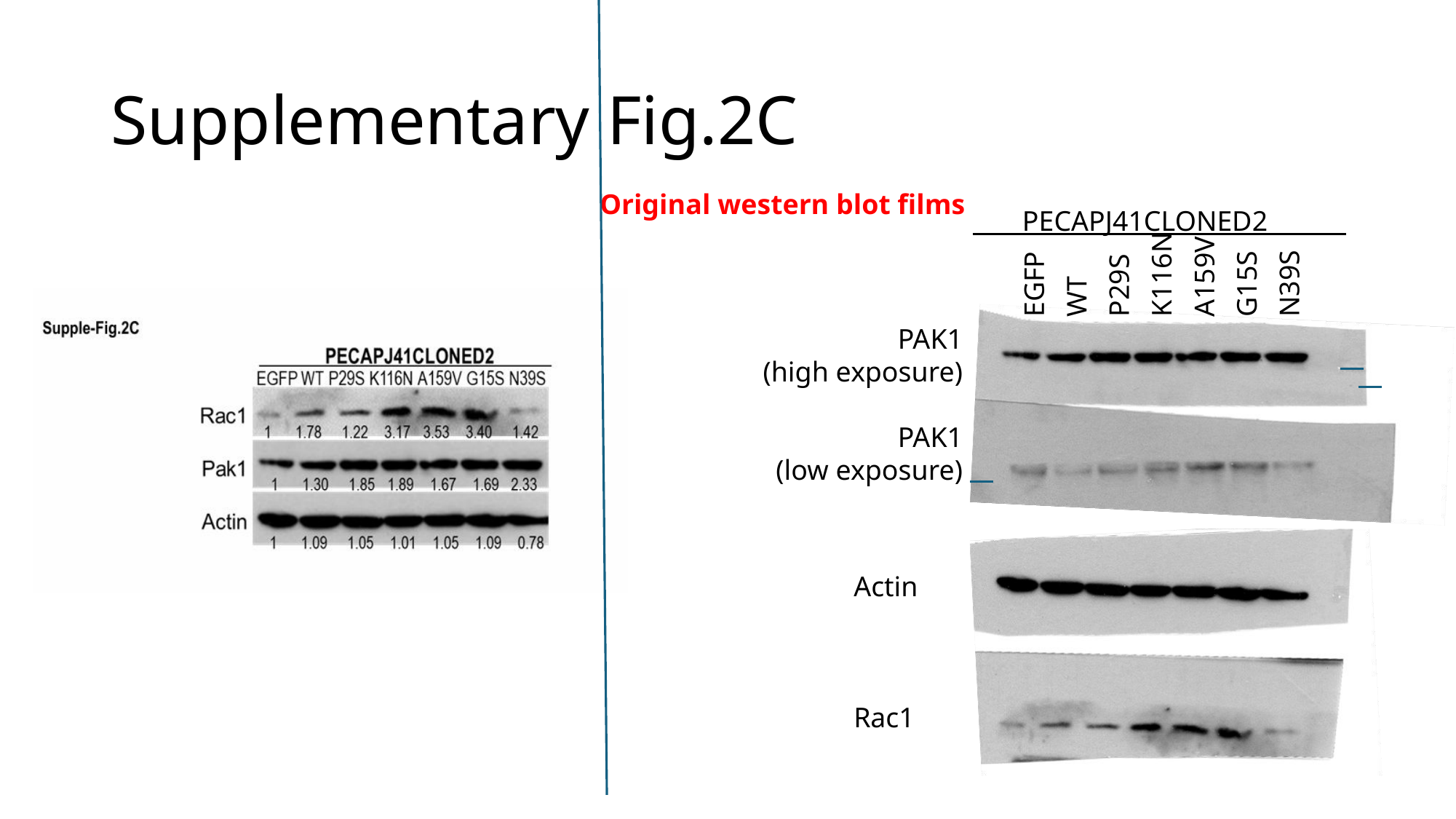

# Supplementary Fig.2C
EGFP
WT
P29S
K116N
A159V
G15S
N39S
Original western blot films
 PECAPJ41CLONED2
PAK1
(high exposure)
PAK1
(low exposure)
Actin
Rac1

## Slide 5
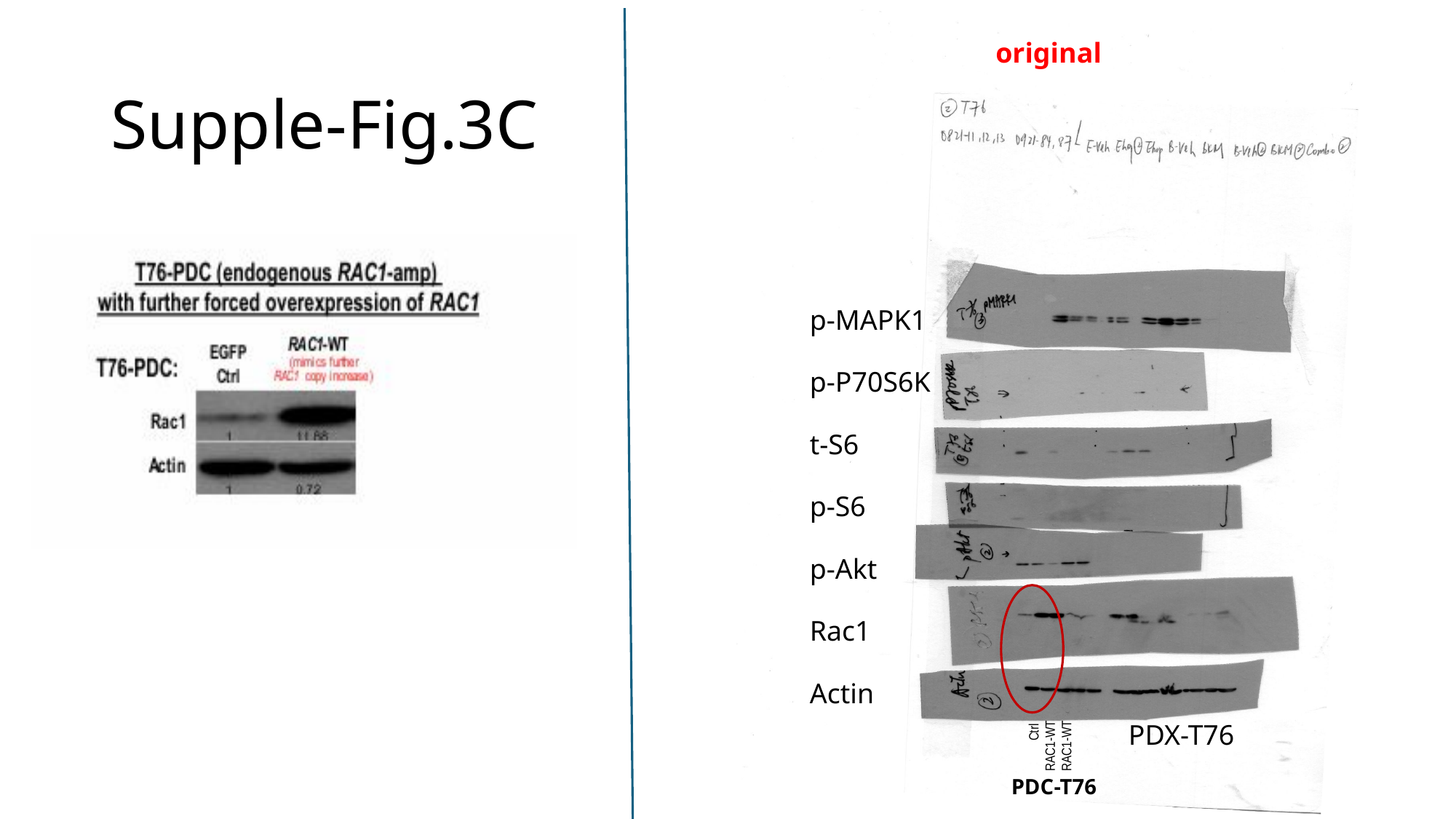

original
# Supple-Fig.3C
p-MAPK1
p-P70S6K
t-S6
p-S6
p-Akt
Rac1
Actin
Ctrl
RAC1-WT
RAC1-WT
PDX-T76
PDC-T76
