# Supplementary material for: RAC1-Amplified and RAC1-A159V Hotspot-Mutated Head and Neck Cancer Sensitive to the Rac Inhibitor EHop-016 In Vivo: A Proof-of-Concept Study
Source: Cancers (Basel). 2025 Jan 23;17(3):361. doi: 10.3390/cancers17030361 (PMC11816149; doi:10.3390/cancers17030361)
Supplement: Supplementary file 1 [file cancers-17-00361-s001.zip › Supplementary Figures.pptx]

## Slide 1
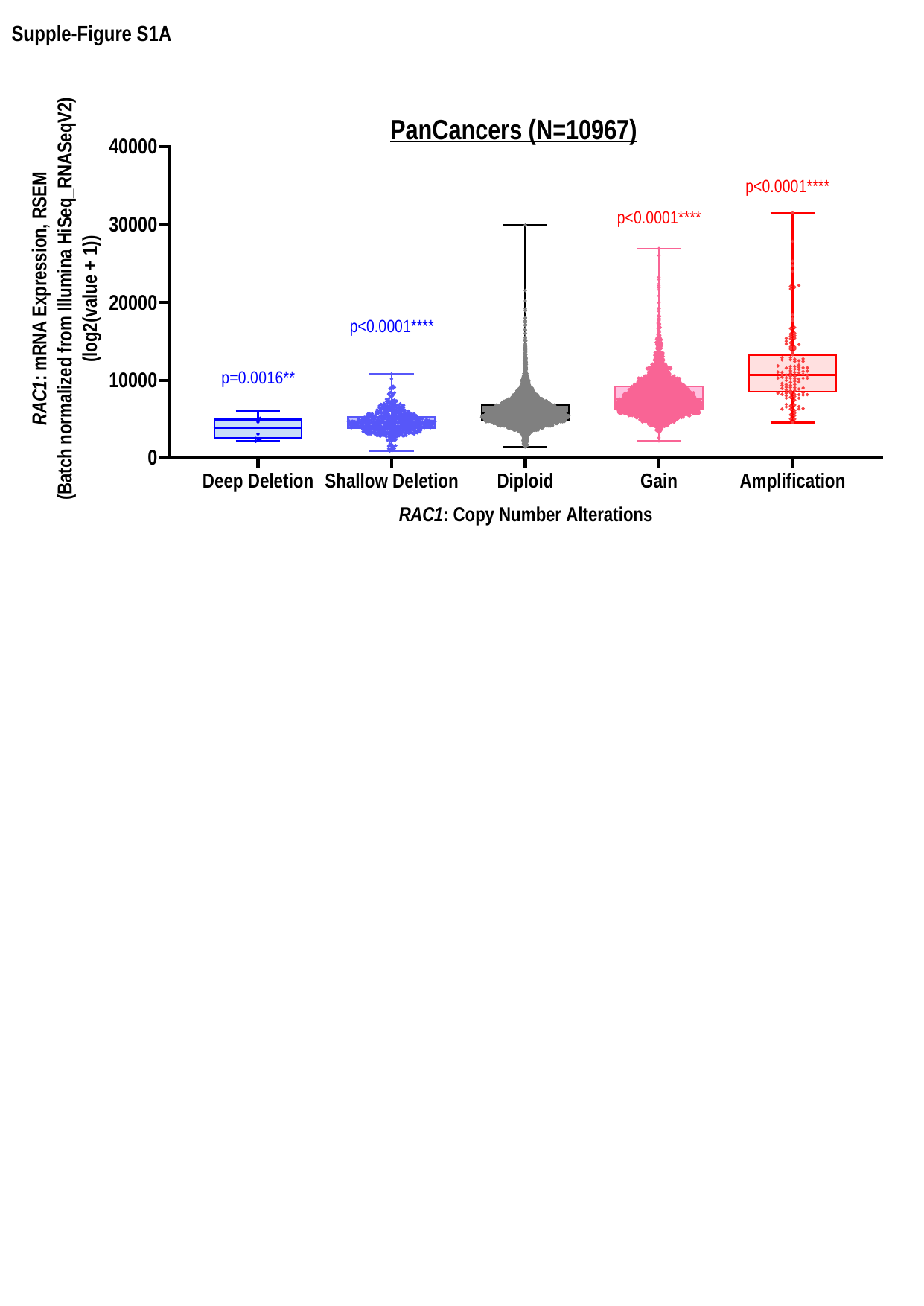

Supple-Figure S1A
PanCancers (N=10967)

## Slide 2
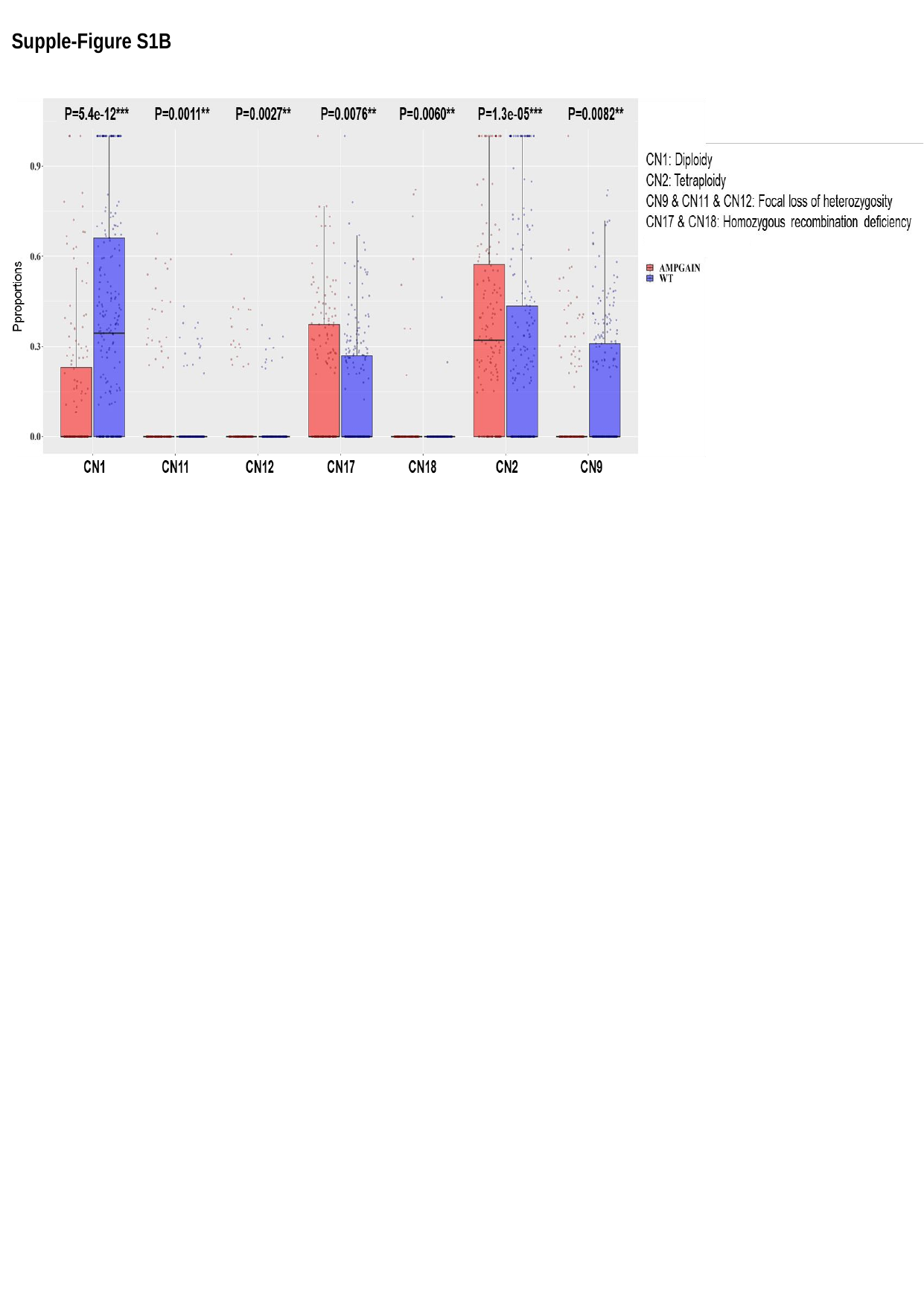

Supple-Figure S1B

## Slide 3
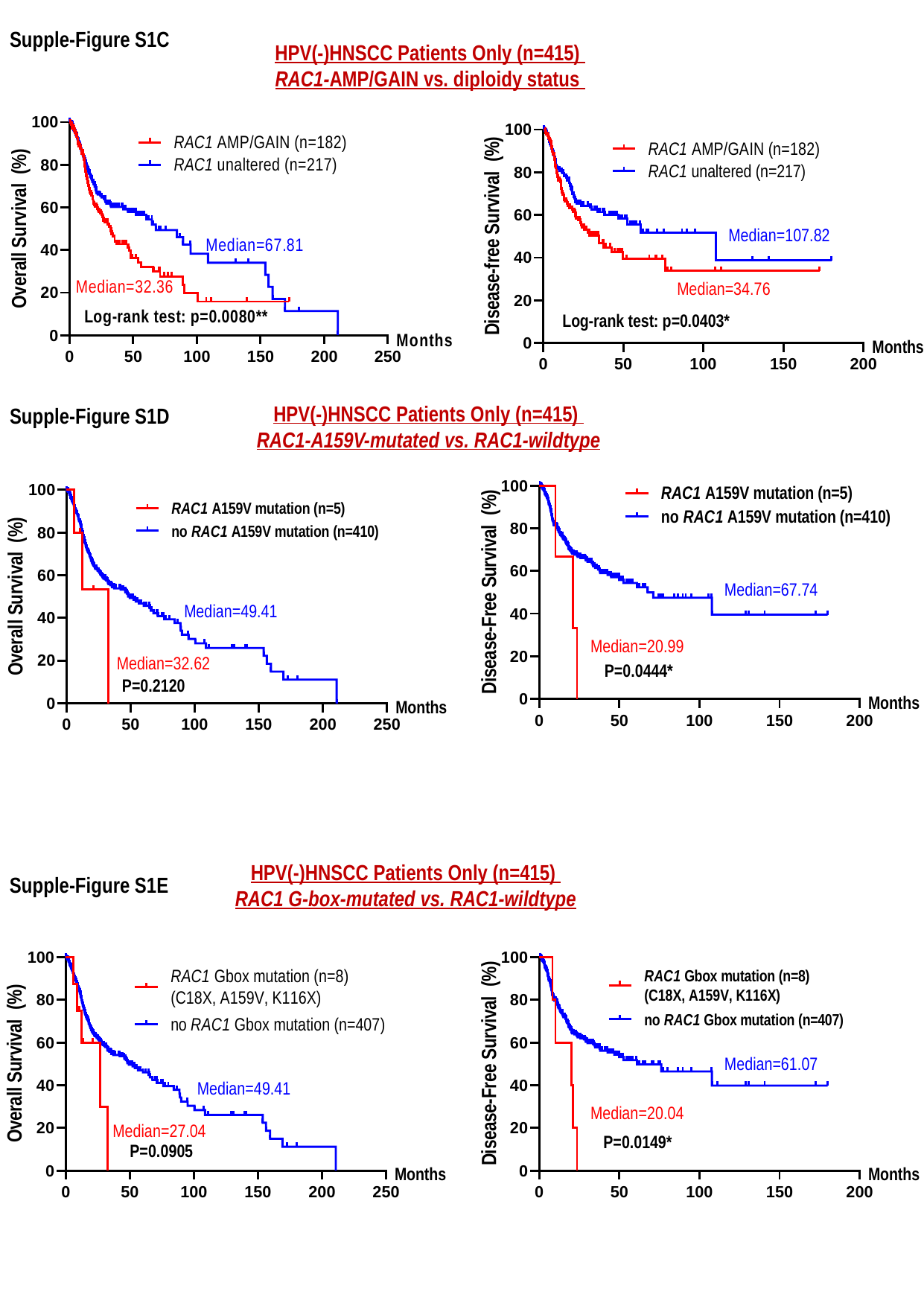

Supple-Figure S1C
HPV(-)HNSCC Patients Only (n=415)
RAC1-AMP/GAIN vs. diploidy status
HPV(-)HNSCC Patients Only (n=415)
RAC1-A159V-mutated vs. RAC1-wildtype
Supple-Figure S1D
HPV(-)HNSCC Patients Only (n=415)
RAC1 G-box-mutated vs. RAC1-wildtype
Supple-Figure S1E

## Slide 4
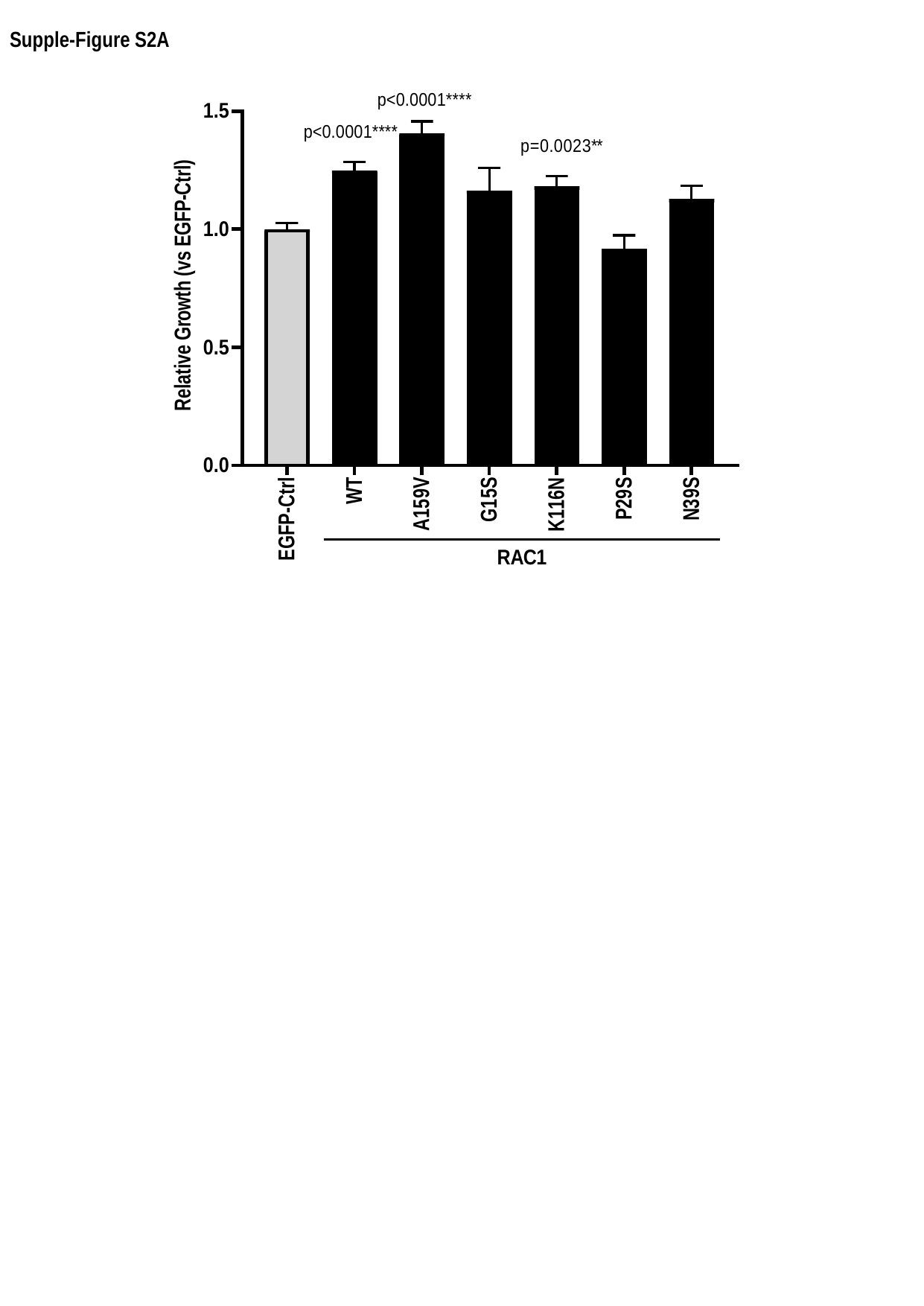

Supple-Figure S2A

## Slide 5
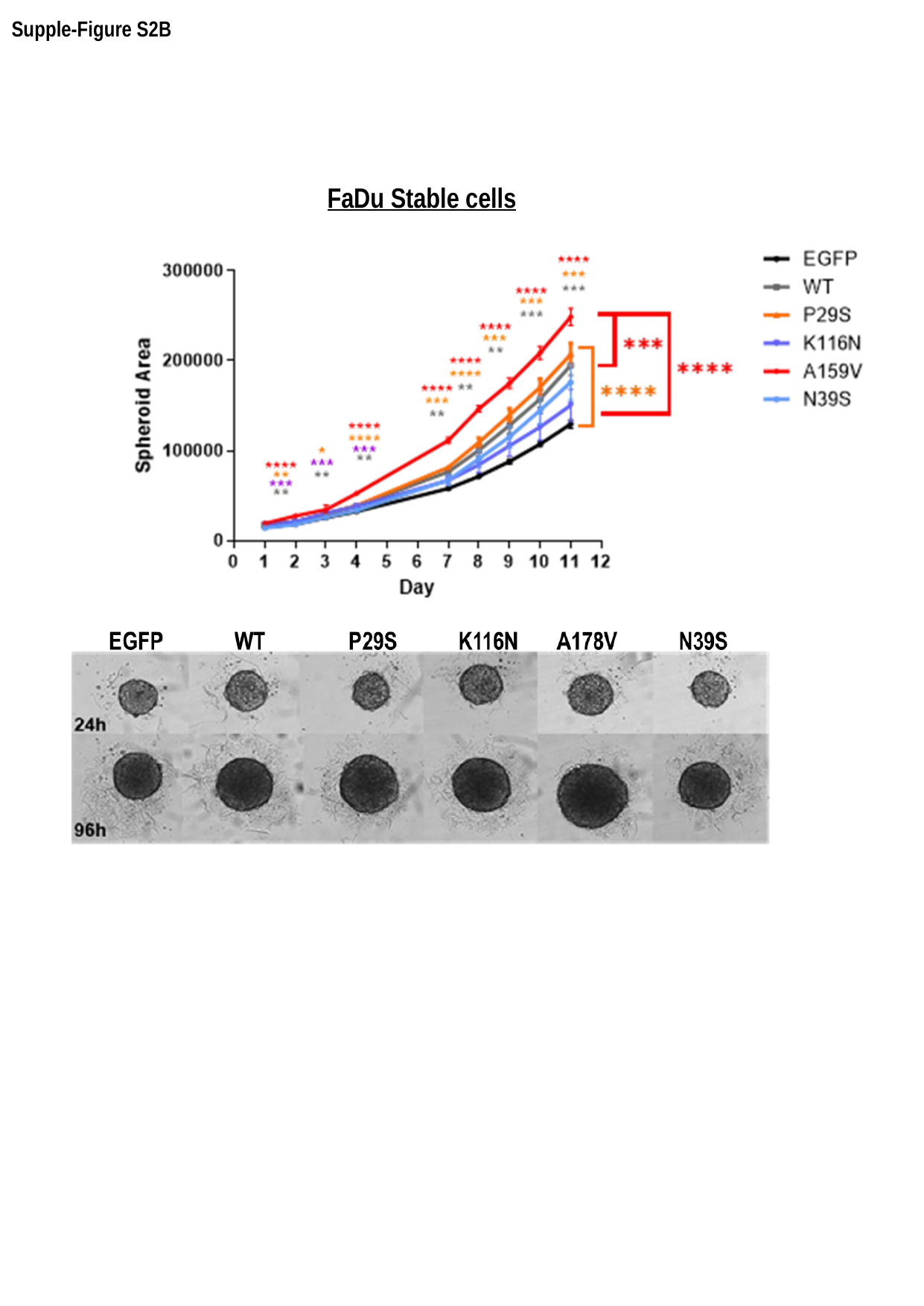

Supple-Figure S2B
FaDu Stable cells

## Slide 6
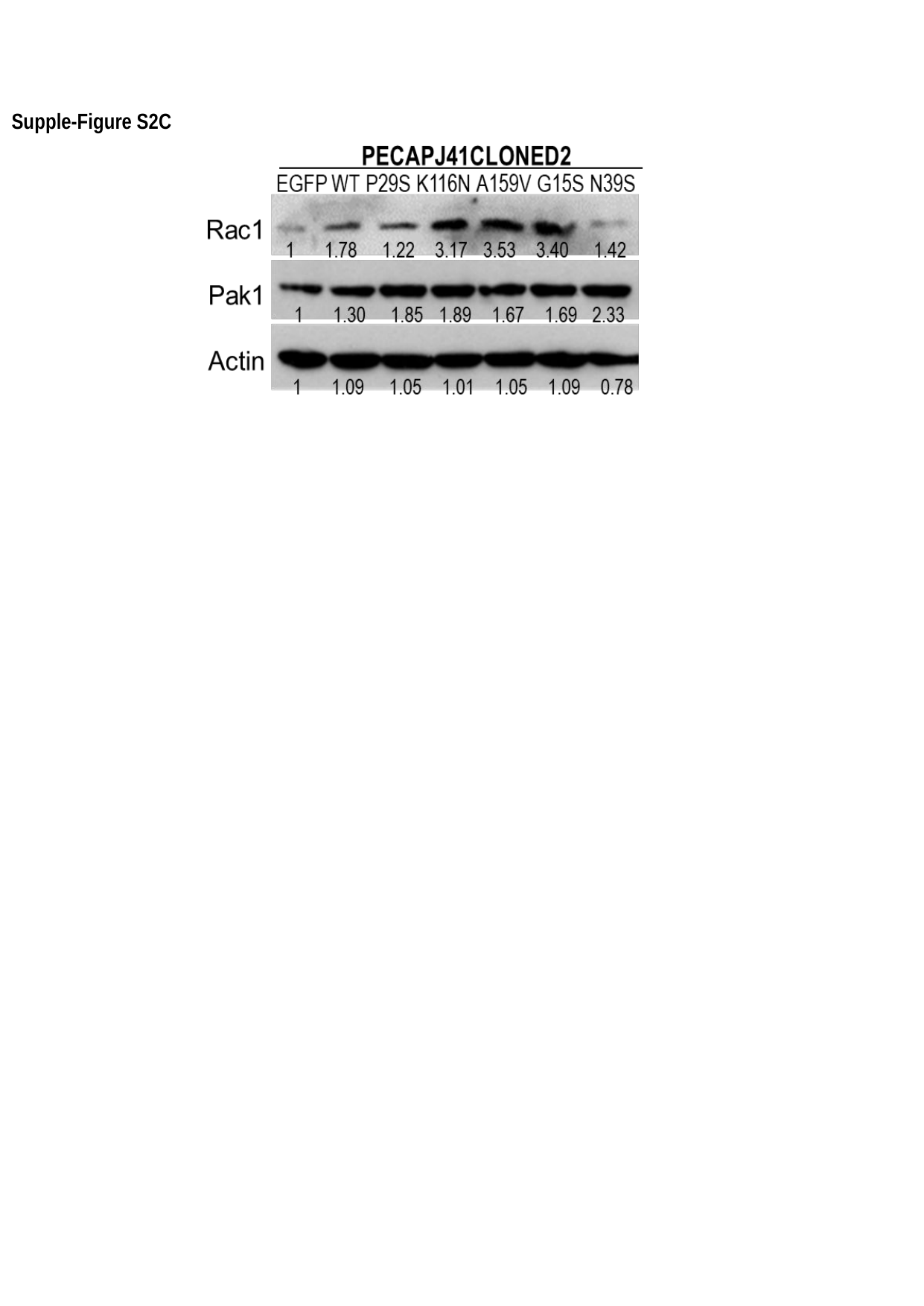

Supple-Figure S2C

## Slide 7
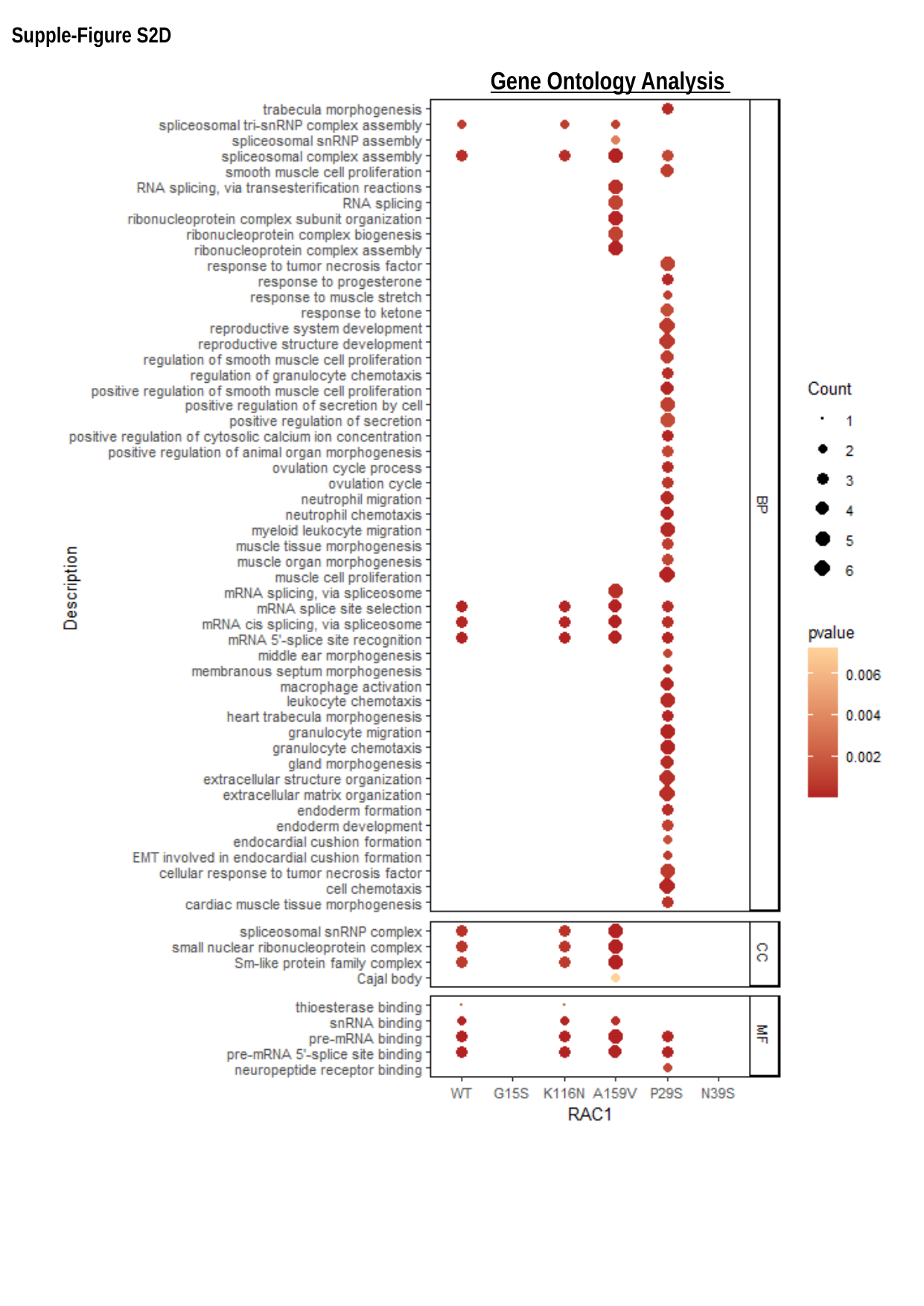

Supple-Figure S2D
Gene Ontology Analysis

## Slide 8
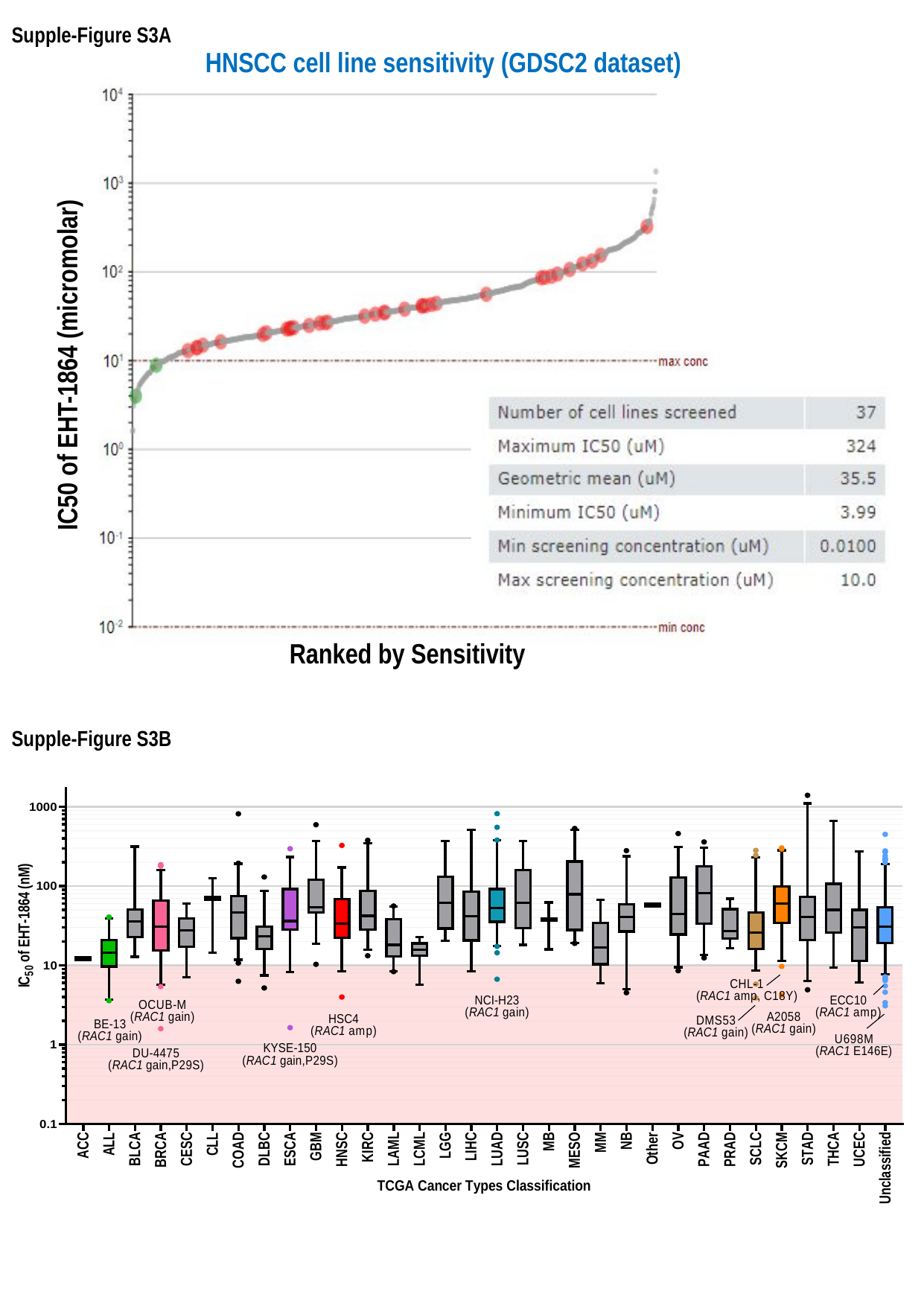

Supple-Figure S3A
HNSCC cell line sensitivity (GDSC2 dataset)
IC50 of EHT-1864 (micromolar)
Ranked by Sensitivity
Supple-Figure S3B

## Slide 9
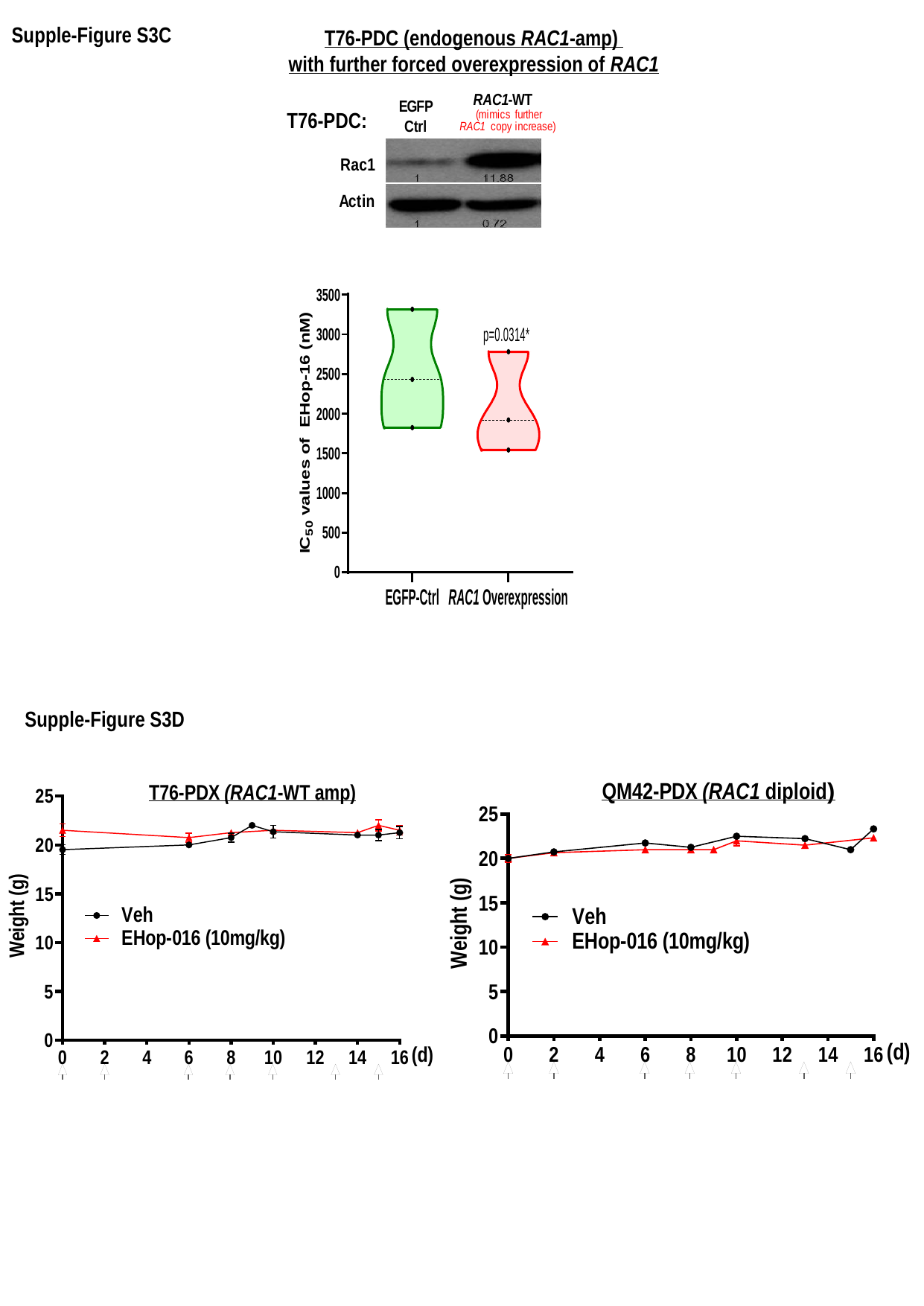

Supple-Figure S3C
T76-PDC (endogenous RAC1-amp)
with further forced overexpression of RAC1
T76-PDC:
Supple-Figure S3D

## Slide 10
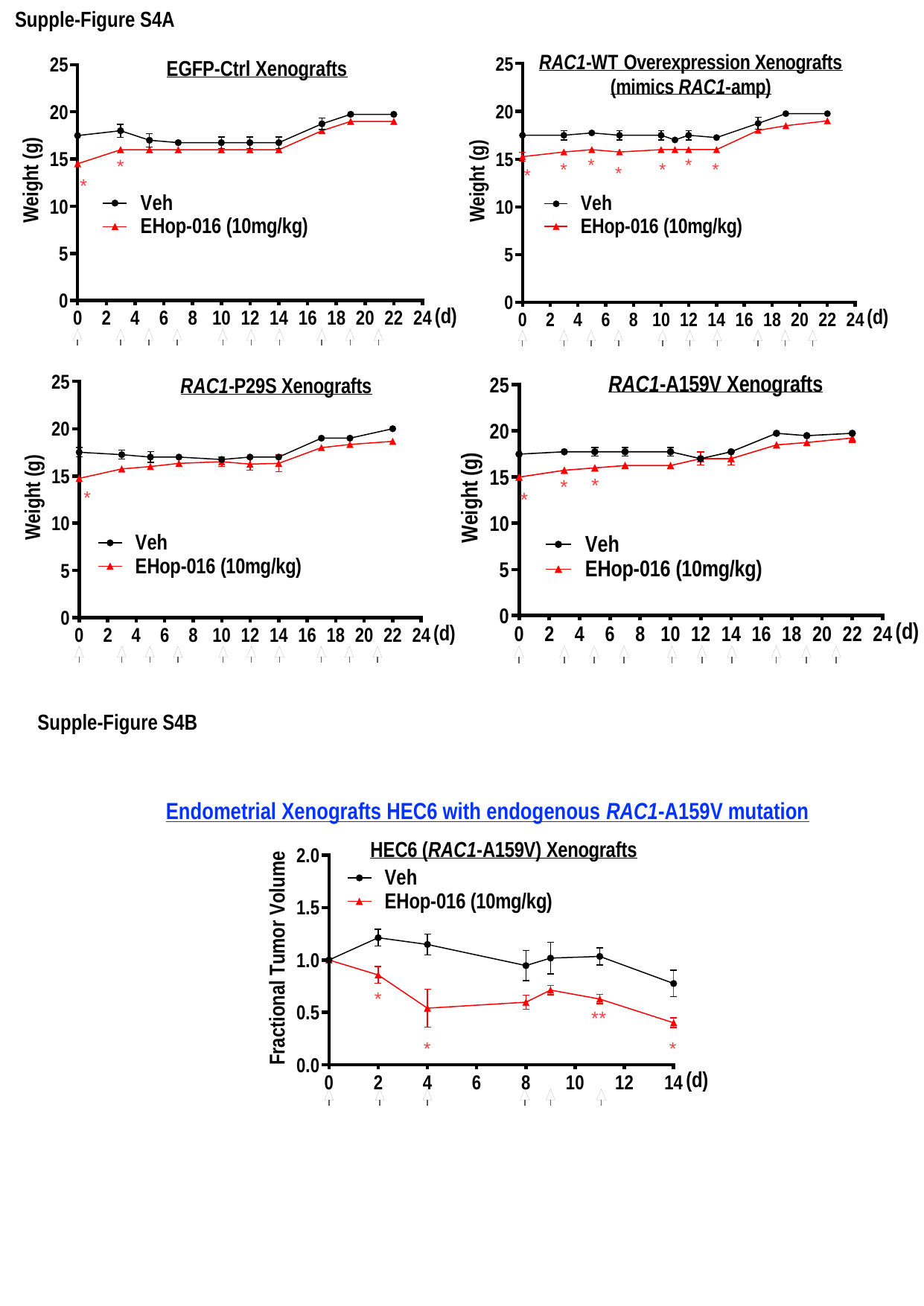

Supple-Figure S4A
Supple-Figure S4B
Endometrial Xenografts HEC6 with endogenous RAC1-A159V mutation

## Slide 11
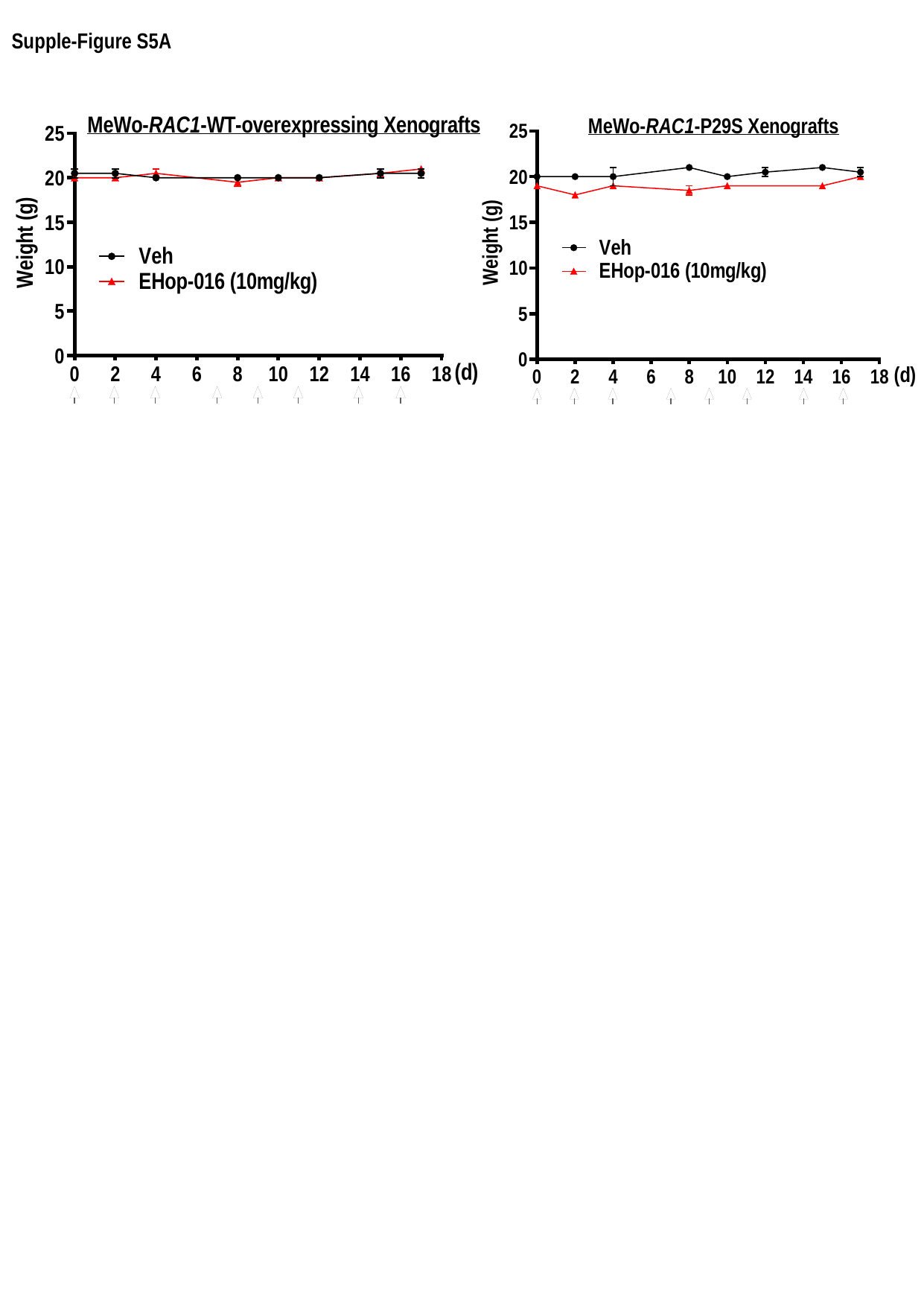

Supple-Figure S5A
